# Quantitative SISCOM assessment for epileptogenic zone localization: insights from a multicenter study comparing two software platforms in temporal lobe epilepsy

**DOI:** 10.3389/fneur.2025.1552774

**Published:** 2025-04-15

**Authors:** Carla Oliveira Young, Brunno Machado de Campos, Edna Marina de Souza, Sergio Querino Brunetto, Maria Julia de Oliveira Santos Gualberto, Leonardo Alexandre-Santos, Marcos Geraldo Merichelo de Oliveira, Marina Koutsodontis Machado Alvim, Fernando Cendes, Elba Etchebehere, Lauro Wichert-Ana, Bárbara Juarez Amorim

**Affiliations:** ^1^Division of Nuclear Medicine, Department of Radiology and Oncology, University of Campinas (UNICAMP), Campinas, Brazil; ^2^Division of Epilepsy, Department of Neurology, University of Campinas (UNICAMP), Campinas, Brazil; ^3^Centre of Biomedical Engineering, University of Campinas (UNICAMP), Campinas, Brazil; ^4^Nuclear Medicine and PET/CT Laboratory, Department of Medical Imaging, Haematology and Clinical Oncology, Ribeirão Preto Medical School, University of São Paulo, Ribeirão Preto, Brazil

**Keywords:** SISCOM, SPECT, quantitative analysis, temporal lobe epilepsy, pharmacoresistant epilepsy, epileptogenic zone

## Abstract

**Introduction:**

Pharmacoresistant epilepsy affects around one-third of individuals with epilepsy, requiring precise diagnosis, particularly in cases where surgical resection of the epileptogenic zone (EZ) is an option. Functional imaging techniques, such as ictal-interictal subtraction SPECT coregistered to MRI (SISCOM), have proven useful in pre-surgical evaluation by improving EZ localization accuracy. However, the widespread use of SISCOM is limited by the high costs and technical complexity of commercial software. Statistical Parametric Mapping (SPM) has been demonstrated to be a viable alternative for SISCOM analysis, displaying the potential for cost-effective EZ localization.

**Materials and methods:**

In this retrospective study, we evaluated patients with pharmacoresistant temporal lobe epilepsy from two reference centers of epilepsy in Brazil, who underwent ictal and interictal SPECT imaging as part of their pre-surgical evaluation, achieving favorable outcomes (Engel I or II) after surgical resection. The EZ reference standard was determined according to anatomopathological findings and good clinical outcomes. SISCOM was performed using a semi-automated approach with Statistical Parametric Mapping (SPM) and a proprietary software – Analyze. Data from each method were compared to the EZ reference standard and classified as concordant, partially concordant, or discordant.

**Results:**

We included 20 patients, 14 (70%) with left temporal lobe epilepsy and six (30%) with right temporal lobe epilepsy. Hippocampal sclerosis was the most common pathology (80%). Both SPM and Analyze were concordant with the EZ reference standard in 14 cases (70%), showing no difference in sensitivity between the methods. However, SPM generated smaller, more localized clusters, while Analyze produced larger clusters with broader spatial coverage. Concordance between the two methods was poor (Kappa = 0.0179), reflecting methodological differences.

**Conclusion:**

This study evidences technical differences between SISCOM performed with SPM and Analyze, but with similar sensitivity (70%) for EZ localization. Further studies with larger sample sizes are required to confirm these findings. The data presented here suggest that SISCOM-SPM, due to its rapid and semi-automated workflow, may offer a practical and accessible alternative to proprietary software for epilepsy surgical planning.

## 1 Introduction

Pharmacoresistant epilepsy affects around one-third of individuals with epilepsy (1). This condition is defined by the failure to achieve seizure control, even after the use of at least two appropriately selected and well-tolerated antiepileptic drugs (1). For these patients, surgical resection of the epileptogenic zone (EZ) is the most effective therapeutic option to achieve seizure freedom (2). However, the precise localization of the EZ remains significantly challenging, particularly in patients with normal or inconclusive magnetic resonance imaging (MRI) (3). Thus, pharmacoresistant epilepsy requires considerable clinical attention, demanding advanced diagnostic and therapeutic strategies (1).

In this context, functional neuroimaging techniques, such as single-photon emission computed tomography (SPECT), have proven to be valuable tools in the pre-surgical evaluation of patients with pharmacoresistant epilepsy, complementing structural MRI findings (3, 4). SPECT, particularly when performed during a seizure (ictal SPECT), assists in identifying the EZ by highlighting increased cerebral perfusion. Generally, cerebral SPECT images are analyzed visually, presenting a diagnostic sensitivity for temporal lobe epilepsy of approximately 97% for ictal SPECT and 44% for interictal SPECT (5). However, visual analysis can often be challenging (6), and an accurate definition of the EZ is crucial (7).

The subtraction ictal-interictal SPECT coregistered to MRI (SISCOM) technique optimizes this process by allowing a direct comparison between ictal (hyperperfused) and interictal (hypoperfused) images, thereby increasing sensitivity and specificity in detecting the EZ (7–11). SISCOM images have higher spatial resolution than ictal or interictal images individually and can accurately localize the EZ, even in patients with focal cortical dysplasia and a normal MRI (12, 13). Studies such as Foiadelli et al. (4) have demonstrated the efficacy of SISCOM, particularly in pediatric epilepsy, assisting in surgical interventions even in cases with inconclusive MRI findings. Additionally, some studies reinforce the role of SISCOM in epilepsy surgery, highlighting improved postoperative outcomes (14).

Despite its clinical value, the widespread use of SISCOM is limited by the high costs and technical complexity of the commercial software required for its analysis (15). Few studies have developed accessible tools to replicate SISCOM’s functionalities at a lower cost, highlighting the need for different solutions (8, 10).

As an alternative, Statistical Parametric Mapping (SPM) is a free software well-documented in the literature for performing cerebral quantification (16–18). Integrating SISCOM with Statistical Parametric Mapping (SPM) has improved image quantification and provided a cost-effective platform (4, 19).

The present authors have already used SPM for performing SISCOM in a previous scientific study, and this approach was designated as SISCOM-SPM (20). The aim of the present study was to apply SISCOM-SPM and compare it with another well-established proprietary software for localizing the EZ. We conducted a multicenter study across two major national reference centers in epilepsy.

## 2 Materials and methods

This is a retrospective, multicentre study involving patients from the University of Campinas Clinical Hospital and the Ribeirão Preto Medical School Clinical Hospital who had previously undergone ictal and interictal cerebral SPECT imaging as part of epilepsy follow-up. Both participating institutions have expertise in these quantitative analyses, including SISCOM-SPM and a proprietary software named Analyze.

This study was approved by the Research Ethics Committee of the Faculty of Medical Sciences of the University of Campinas (UNICAMP). The protocol was registered under the CAAE: 36549220.9.1001.5404.

### 2.1 Inclusion and exclusion criteria

Inclusion criteria for this study were as follows: patients with pharmacoresistant epilepsy, unifocal temporal epilepsy; those who previously undergone ictal and interictal cerebral SPECT studies as part of the routine investigation for the EZ; those who agreed to provide informed consent; submitted to surgical resection; seizure-free or almost seizure-free outcome after surgery (Engel I and II).

Exclusion criteria consisted of extratemporal or multifocal temporal epilepsy, not having both ictal and interictal cerebral SPECT studies available, and refusal to participate in the study or to provide informed consent.

As all patients underwent surgery for EZ resection, the reference standard for EZ localization was defined by the location of anatomopathological findings indicating areas consistent with epileptogenic lesions alongside favorable patient outcomes (Engel I or II). This is considered the gold standard for EZ localization (13).

### 2.2 Patients selection

We analyzed the medical records of patients who underwent cerebral SPECT for epileptogenic focus investigation between 2015 and 2019, who proceeded to surgical resection of the EZ and demonstrated a favorable clinical outcome, remaining seizure-free or with rare seizures (Engel I and II). Patients were selected from two national reference institutions, the University of Campinas and the Ribeirão Preto Medical School, in Brazil.

### 2.3 Patients preparation for interictal SPECT imaging

All patients remained in a low-light, quiet room for 15 min, with permanent intravenous access through a butterfly connected to a catheter with saline solution. While at rest, 1110 MBq (30 mCi) of ^99m^Tc-ECD were injected. The patients rested for another 10 min before the SPECT acquisition. EEG monitoring was not performed during interictal SPECT acquisition, but no seizures were observed clinically.

### 2.4 Patients preparation for ictal SPECT imaging

All patients were monitored with a long-term video-EEG. The antiseizure medication was reduced in selected cases to increase the chance of epileptic seizures. Patients rested while continuous video-EEG was recorded. All patients remained with permanent intravenous access through a butterfly connected to a catheter with a saline solution. A syringe with the radiotracer was attached to the catheter and protected with a lead shield to ensure a fast injection. Upon seizure onset, around 1,110 MBq (30 mCi) of ^99m^Tc-ECD were quickly injected. EEG and video recordings confirmed seizures. SPECT images were acquired 30–90 min after seizure cessation and patients’ symptoms. For the five patients from the Campinas cohort, radiotracer injection occurred between 13 and 24 s after seizure onset (mean: 16.6 s), consistent with literature recommendations for optimal EZ localization (13). Injection time data were not available for the Ribeirão Preto cohort due to retrospective data limitations.

### 2.5 Brain SPECT acquisition

SPECT was performed using a Symbia T2 SPECT/CT system (Siemens, Erlangen, Bayern, Germany) with a high resolution, low energy, two-head collimator. The SPECT images were acquired, with photopeak centered at 140 keV and 15% window, 128 × 128 matrix, 2.67 zoom (which could be variable), and 64 views for each head (37 s per view). Raw data were reconstructed with 3D OSEM (17 intersections, 16 subsets), 7.65 mm Gaussian filter and CT attenuation correction. Images were displayed in transaxial, coronal, and sagittal planes for visual analysis.

### 2.6 MRI acquisition

The MRI epilepsy protocol was conducted on a 3 Tesla Philips Achieva scanner and included: (1) Coronal images perpendicular to the hippocampus: T1-weighted “inversion recovery” (voxel of 0.64×0.64×3 mm^3^, gap of 0.3 mm, 45 slices, TR 3,550 ms, TE 15 ms, IR delay of 400 ms); (2) Axial images parallel to the hippocampus: DWI (voxel of 1.19×1.19×4 mm^3^, 28 slices, TR 3,474 ms, TE 70 ms, max b-factor of 1,000 s/mm^2^); (3) Additional 3D sequences aligned to the corpus callosum: 3D T1-weighted image (1 mm isotropic voxels, matrix of 240×240, 180 sagittal slices, TR of 7 ms, TE of 3.2 ms), 3D-FLAIR (voxel of 1.2×1.2×1.2 mm^3^, matrix of 180×180 and 300 slices, TR of 1,000 ms, TI of 2,400 ms), T2WI 3D (1 mm isotropic voxels, matrix of 250×250, 327 slices, TR of 2000 ms and TE of 364 ms).

### 2.7 SPECT visual analysis

Two experienced nuclear medicine physicians with over 20 years of experience in nuclear medicine brain images performed the analysis. These two nuclear medicine physicians evaluated the images in consensus, looking for focal areas of hyper or hypoperfusion and comparing both cerebral hemispheres. They also compared brain perfusion with the cerebellar perfusion. Nuclear medicine physicians were aware of clinical and electroencephalographic findings. The EZ was defined as a focal area of hyperperfusion in the ictal SPECT images, which became hypoperfused or normally perfused in interictal SPECT images. When there was more than one hyperperfused area, we correlated the findings with the time injection and ictal semiology to define the EZ. The other areas were considered as propagation areas.

### 2.8 Subtraction ictal-interictal SPECT co-registered to MRI with SPM (SISCOM-SPM)

A trained physician performed the ictal and interictal subtractions using the Statistical Parametric Mapping (SPM) software, version 12 (SPM12) (Wellcome Department of Imaging Neuroscience, University College London, UK)^1^ in Matlab (MathWorks, Natick, MA, USA) (21).

After acquiring and reconstructing interictal and ictal images, they were converted from DICOM to Analyze format using MRIcro software. Both ictal and interictal images in the Analyze format were loaded into SPM12, which runs in Matlab software.

The images were subsequently realigned using the anterior commissure as a reference. In the registration step, ictal and interictal images were registered using routines based on Mutual Information maximization algorithms. Both images were taken as target and source in two different registrations. The registered images were realigned, and the mean intensity value calculated, normalizing the uptake levels toward the whole brain. The registered images’ positional correspondence and mean were checked in SPM12. After establishing that all areas were correspondent, the subtraction was done. To obtain the cerebral perfusion differences, the transformed and normalized interictal SPECT image was subtracted voxel by voxel from the ictal SPECT image using the LCN12 subtract routine (21). The difference image was transformed into a z-score using the mean and standard deviation (SD) of the differences in all brain voxels (22). All voxels exceeding two z-scores were considered significant. Usually, a hyperperfused region appears as a cluster, which is a set of voxels.

The subtraction process with SPM took approximately 15 min.

### 2.9 Subtraction ictal-interictal SPECT co-registered to MRI with Analyze (SISCOM-Analyze)

SISCOM was executed using the ANALYZE © 10.0 Software (AnalyzeDirect, Inc., Overland Park, Kansas, USA), applying a methodology adapted from prior research (23). Subtraction was carried out by subtracting the interictal SPECT scan from the ictal SPECT, transforming the difference in the signal into z-score maps, calculated from the mean and standard deviation across all brain voxels, producing images that reflected changes in regional cerebral blood flow (rCBF). This quantitative data matrix was then fused with the patient’s MRI to integrate functional and anatomical insights within a single image. After functional overlay, only regions with significant changes in rCBF—defined as greater than two standard deviations above or below the mean—were displayed.

The subtraction process with Analyze typically takes approximately 60 min when performed by an expert, whereas it can exceed 2 h for less experienced individuals. While this reflects general practice, future studies are needed to accurately quantify the time required under different levels of expertise.

### 2.10 EZ analysis with SISCOM-SPM and SISCOM-Analyze

Specific parameters were adopted to investigate the localization of the epileptogenic zone, including the voxel with the highest intensity and the largest clusters acquired through SISCOM performed using both methods: (a) EZ would be the highest intensity cluster (Z score); (b) EZ would be the largest cluster (number of voxels); (c) EZ would be the second largest cluster (number of voxels); and (d) EZ would be the third-largest cluster (number of voxels).

### 2.11 SISCOM analyses vs. EZ reference standard

**Figure 1:**
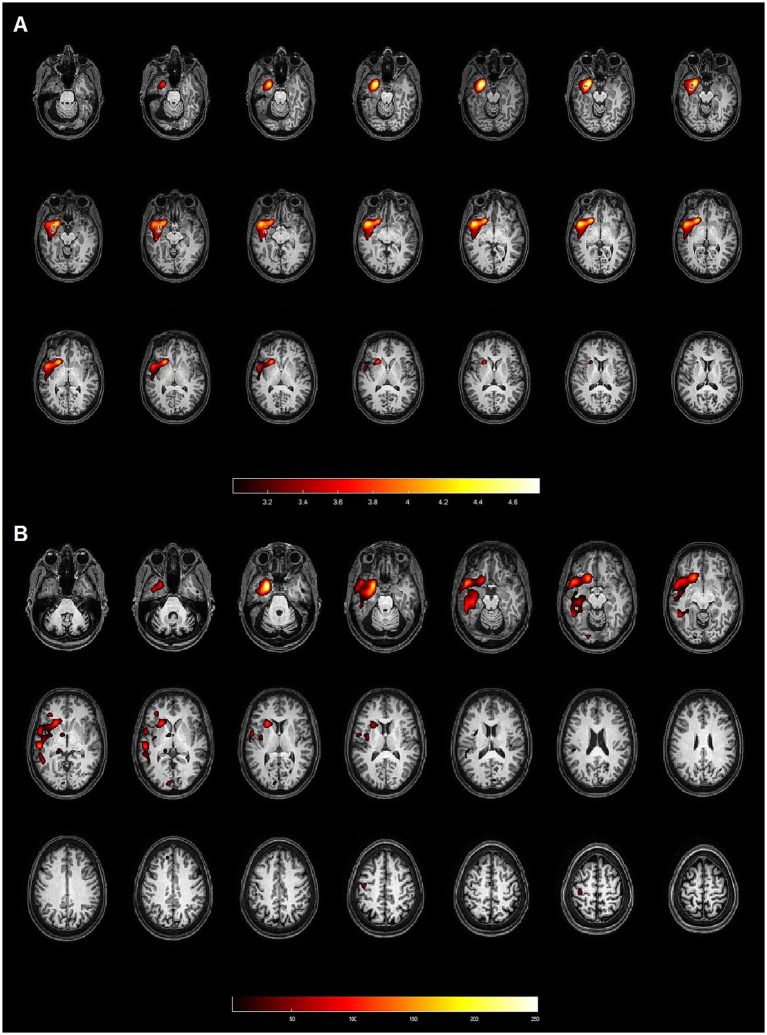
Patient 4: 50-year-old female with left hippocampal sclerosis. Both methods (SPM and Analyze) demonstrated concordance with the gold standard, identifying the EZ in the left temporal lobe. **(A)** Axial slices from the SISCOM analysis using SPM, showing the largest cluster and the voxel with the highest intensity located in the left temporal lobe, concordant with the EZ. **(B)** Axial slices from the Analyze software, also identifying the largest cluster and the voxel with the highest intensity in the left temporal lobe, both concordant with the EZ.

**Figure 2:**
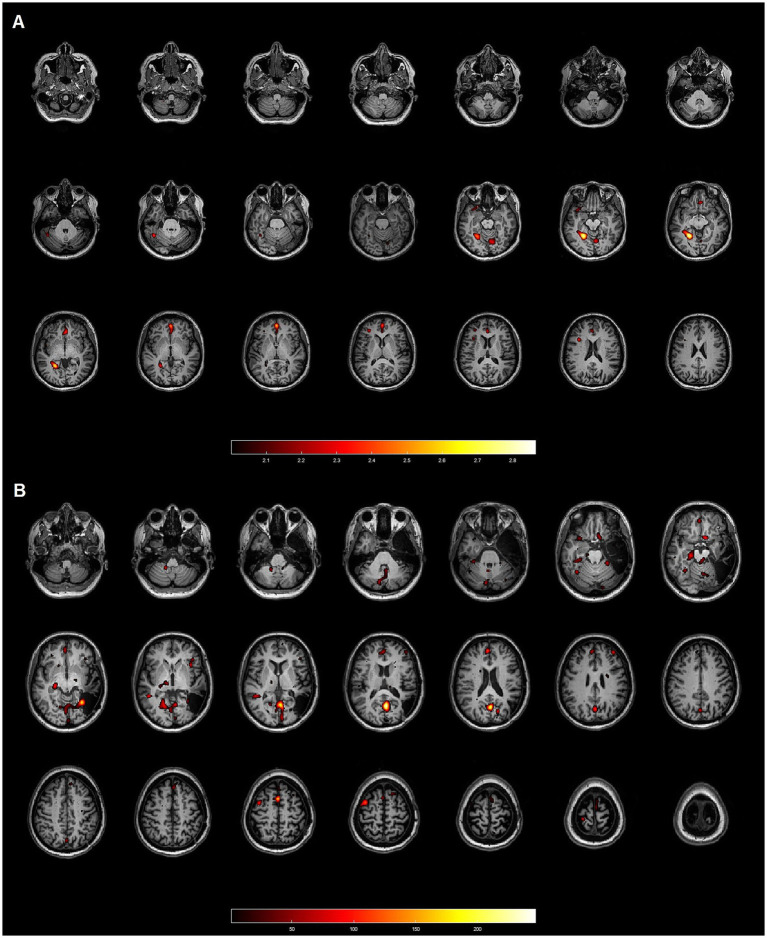
Patient 17: 45-year-old female with focal cortical dysplasia (FCD) IB in the right temporal lobe. Neither method demonstrated concordance with the gold standard. **(A)** Axial slices from the SISCOM analysis using SPM, showing multiple clusters discordant with the EZ, which was located in the right temporal lobe. **(B)** Axial slices from the Analyze software, also displaying multiple clusters inconsistent with the EZ in the right temporal lobe.

The location of the EZ identified by both methods, SISCOM-SPM and SISCOM-Analyze, was compared to the reference standard and categorized as follows:

- Concordant: when there was an overlap between the hyperperfused area and the EZ (Figure 1);
- Partially concordant: when the hyperperfused area was adjacent or very close to the EZ;
- Discordant: when the hyperperfused area was distant from the EZ (Figure 2).

### 2.12 Statistical analysis

Descriptive analysis was performed for categorical variables using frequency tables and quantitative variables using measures of central tendency and dispersion. Agreement between methods (SISCOM-Analyze and SISCOM-SPM with the EZ reference standard) was evaluated using the Kappa coefficient, with values classified as poor (<0.40), moderate (0.40–0.75), or excellent (>0.75). Mann–Whitney and Fisher’s exact tests were applied to compare groups, and Spearman’s rank correlation coefficient was used to calculate the numerical variables. The level of significance adopted was 5%.

In some analyses, cases classified as discordant and partially concordant were grouped into a non-concordant category to facilitate statistical evaluation and simplify interpretation.

## 3 Results

We analyzed the medical records of 97 individuals. Among them, we included five patients from the University of Campinas and 15 patients from Ribeirão Preto School, who had ictal and interictal SPECT scans, and proceeded to surgical resection of the EZ and had a favorable postoperative outcome (Engel I and II). Therefore, we included 20 patients from both institutions in total. Their mean age was 42.5 ± 12.2 years (median: 44.5 years; range: 10.0–58.0 years). Eleven patients (55.0%) were female and nine (45.0%) were male. All patients had temporal lobe epilepsy, and based on the reference standard, six (30.0%) had right temporal lobe epilepsy, and 14 (70.0%) had left temporal lobe epilepsy.

According to anatomopathological findings, 16 (80.0%) had hippocampal sclerosis, one (5.0%) focal cortical dysplasia and three (15.0%) other pathologies (astrogliosis, grade 1 ganglioglioma and fibrillar gliosis). Table 1 summarizes the demographic data, gold standard localization, anatomopathological findings, and post-surgical outcomes (Engel classification) of the 20 patients included in the study.

**Table 1:** Patients’ clinical and anatomopathological characteristics.

Patient No.	Age	Gender	Gold standard	Anatomopathological finding	Engel
1	51	Female	Left temporal	Left hippocampal sclerosis	I-A
2	53	Female	Left temporal	Left hippocampal sclerosis	I-A
3	37	Female	Left temporal	Left hippocampal sclerosis	I-A
4	50	Female	Left temporal	Left hippocampal sclerosis	I-A
5	10	Female	Left temporal	Left hippocampal sclerosis	I-A
6	52	Male	Right temporal	Right hippocampal sclerosis	I-D
7	44	Male	Right temporal	Right hippocampal sclerosis	I-A
8	51	Male	Left temporal	Left hippocampal sclerosis	II-A
9	58	Male	Right temporal	Right hippocampal sclerosis	I-A
10	28	Male	Left temporal	Left hippocampal sclerosis	I-A
11	52	Female	Left temporal	Left hippocampal sclerosis	I-B
12	27	Female	Left temporal	Left hippocampal sclerosis	I-A
13	40	Male	Right temporal	Right hippocampal sclerosis	I-C
14	34	Female	Left temporal	Left hippocampal sclerosis	I-A
15	51	Male	Left temporal	CA1 fibrillary gliosis	I-B
16	42	Female	Left temporal	Ganglioglioma	I-D
17	45	Female	Right temporal	Focal cortical dysplasia IB	I-A
18	57	Male	Left temporal	Left hippocampal sclerosis	II-A
19	29	Male	Right temporal	Astrogliosis	I-A
20	39	Female	Left temporal	Left hippocampal sclerosis	I-D

### 3.1 SISCOM results

The mean total cluster volume was 29,778.1 ± 17,920.2 mm^3^ using SPM and 17,386.8 ± 3,969.9 voxels using Analyze.

The mean number of clusters in hippocampal sclerosis patients was 11.8 (SD = 12.3) and 34.0 in the other pathologies (SD = 20.7), and this difference had statistical significance (*p* = 0.0252).

Table 2 presents the highest voxel intensity, total number of clusters, and the volumes of the three largest clusters (in mm^3^) identified by the SPM and Analyze methods.

**Table 2:** Quantitative SISCOM results.

Patient No.	SPM					Analyze				
	Highest intensity	Number of clusters	1st cluster (mm^3^)	2nd cluster (mm^3^)	3rd cluster (mm^3^)	Highest intensity	Number of clusters	1st cluster (mm^3^)	2nd cluster (mm^3^)	3rd cluster (mm^3^)
1	3.46	9	803.0	304.6	110.8	249	79	18,157.5	10,283.6	3,618.0
2	3.70	16	494.0	125.0	82.0	250	88	6,048.0	4,704.8	3,675.4
3	4.63	6	8,566.0	3.0	3.0	254	120	15,990.8	4,363.9	3,125.3
4	4.75	7	22,522.7	3.5	0.8	252	23	58,276.1	1,420.9	948.4
5	5.12	6	17,191.1	1,160.0	100.0	254	95	43,173.0	8,339.6	2,322.0
6	2.92	4	17,816.9	7,610.0	6,712.0	255	12	30,240.0	13,864.5	9,072.0
7	3.32	8	20,311.0	11,723.0	3,116.0	251	48	16,544.3	12,693.4	11,724.8
8	3.63	12	54,264.1	6,893.0	1,360.0	253	21	63,723.4	14,738.6	4,765.5
9	3.55	11	29,354.9	2,246.0	1,189.0	254	30	25,882.9	6,183.0	2,298.4
10	3.10	13	9,906.0	6,887.0	4,344.0	254	86	13,510.1	8,066.3	3,688.9
11	4.79	6	55,616.5	3,021.0	1,069.0	254	31	43,135.9	3,705.8	2,862.0
12	2.64	6	24,269.2	7.0	6.0	254	21	24,094.1	7,435.1	1,022.6
13	3.48	56	48,057.0	1,835.0	1,576.0	254	44	26,038.1	16,166.3	6,584.6
14	3.72	14	11,163.0	7,469.0	2,450.0	255	95	16,048.1	9,510.8	4,475.3
15	3.48	44	42,538.1	4,546.9	3,983.9	255	63	40,516.9	6,179.6	2,912.6
16	3.83	10	29,206.1	3,377.0	1,071.0	255	93	11,623.5	10,543.5	5,194.1
17	2.87	25	5,531.0	3,663.0	1,610.0	247	66	22,086.0	5,531.6	3,344.6
18	2.68	6	8,464.9	3,552.0	1,837.0	245	52	11,326.5	6,162.8	4,998.4
19	3.71	57	20,035.1	17,460.1	544.0	254	41	26,044.9	16,186.5	6,588.0
20	3.94	8	11,002.0	6,559.0	3,659.0	252	67	9,976.5	8,046.0	3,483.0

Highest intensity cluster – The average intensity was 3.7 ± 0.7 in SPM and 252.6 ± 2.8 in Analyze. The mean number of clusters was 16.2 ± 16.4 in SPM and 58.8 ± 31.0 in Analyze.

Largest cluster – The mean volume of the largest cluster was 21,855.6 ± 16,798.5 mm^3^ in SPM and 26,121.8 ± 16,032.4 mm^3^ in Analyze, with mean intensities of 2.7 ± 0.5 and 49.7 ± 9.2, respectively.

Second largest cluster – The mean volumes were 4,422.2 ± 4,471.7 mm^3^ in SPM and 8,706.3 ± 4,241.7 mm^3^ in Analyze, with mean intensities of 2.5 ± 0.4 and 39.1 ± 11.0, respectively.

Third largest cluster – The mean volumes were 1,741.2 ± 1,815.0 mm^3^ in SPM and 4,335.2 ± 2,594.9 mm^3^ in Analyze, with mean intensities of 2.4 ± 0.4 and 42.0 ± 10.2, respectively.

### 3.2 Patients based analysis

In the 20 patients, SPM identified 14 patients as concordant with EZ, seven by highest intensity and 14 by highest cluster volume with a sensitivity of 70%. The largest cluster also identified all seven patients with the highest intensity (Table 3). Analyze identified EZ in 14 patients, either by the highest intensity or largest cluster (Table 3), with a 70% sensitivity.

**Table 3:** Concordance of SISCOM analyses with the epileptogenic zone (EZ) reference standard.

Patient		SPM				Analyze			
No.	Gold standard	Highest intensity voxel	1st cluster (mm^3^)	2nd cluster (mm^3^)	3rd cluster (mm^3^)	Highest intensity voxel	1st cluster (mm^3^)	2nd cluster (mm^3^)	3rd cluster (mm^3^)
1	Left temporal	D	C	PC	PC	C	C	C	D
2	Left temporal	C	C	C	PC	D	D	C	D
3	Left temporal	C	C	C	D	C	C	D	C
4	Left temporal	C	C	C	C	C	C	D	D
5	Left temporal	C	C	D	PC	C	C	D	C
6	Right temporal	D	C	D	C	D	D	D	C
7	Right temporal	D	D	D	C	PC	D	D	PC
8	Left temporal	D	C	D	D	C	C	D	D
9	Right temporal	D	D	PC	D	C	C	C	D
10	Left temporal	D	D	D	D	C	C	D	D
11	Left temporal	PC	C	D	D	C	C	C	D
12	Left temporal	C	C	C	PC	C	C	C	D
13	Right temporal	C	C	D	D	C	C	D	D
14	Left temporal	D	D	D	D	C	C	D	D
15	Left temporal	D	C	D	D	C	C	D	D
16	Left temporal	PC	C	D	D	C	C	C	PC
17	Right temporal	D	D	D	D	D	D	D	D
18	Left temporal	D	D	D	PC	D	D	D	PC
19	Right temporal	D	C	D	D	C	C	D	D
20	Left temporal	C	C	D	D	D	D	D	C

C, Concordant; PC, Partially Concordant; D, Discordant.

## 4 Discussion

The present study analyzed the free software Statistical Parametric Mapping (SPM) and the proprietary software Analyze for EZ localization in patients with drug-resistant temporal lobe epilepsy from two national reference centers for epilepsy studies. We demonstrated that both software tools used to perform SISCOM have similar capabilities to localize the EZ, with a sensitivity of 70%.

The primary institution has experience with SPM (24, 25) and previously published results from a study featuring 23 patients with pharmacoresistant epilepsy, demonstrating that SISCOM-SPM improved EZ localization by 21.8% compared to visual analysis alone (20). Meanwhile, the participating institution has scientific experience with Analyze software, which has been applied by the team in epilepsy studies (3, 23, 26) as well as for detecting reperfusion in systemic lupus erythematosus (27).

We noticed a low concordance between SISCOM performed with SPM and Analyze regarding the highest intensity voxel (Kappa: 0.0179). Matsuda et al. (10) also recognized significant variability in the performance of different software tools in clinical and research involving multicenter studies. It can be attributed to differences in how each algorithm processes the data, potentially impacting its sensitivity and spatial resolution. In our study, the poor concordance (Kappa = 0.0179) likely reflects intrinsic differences between the software algorithms, including variations in image preprocessing, intensity normalization, z-score thresholding, and spatial smoothing. SPM tends to identify smaller, focal hyperperfused clusters, while Analyze detects broader activation patterns that may also include propagation zones. Future studies incorporating voxel-wise overlap analysis and algorithmic benchmarking could provide deeper insights into these discrepancies and support standardization across platforms.

A significant difference was observed between the methods compared to applicability in clinical practice. Analyze depends on the technician who performs it, with processing steps lasting over 2 h, depending on the professional’s experience. In contrast, the method developed by the researchers of this study using SPM offers a practically automated flow, with processing completed in a few minutes, making it more accessible and efficient in clinical routines. From a clinical perspective, the streamlined workflow of SISCOM-SPM, requiring approximately 15 min for image processing, offers a considerable advantage over the more time-consuming and operator-dependent Analyze software. This difference has practical implications for routine epilepsy centers, where timely decision-making and reproducibility are essential for surgical planning.

In the present study, SISCOM performed with SPM revealed clusters with smaller volumes (mean volume of the largest cluster: 21,855.6 mm^3^ for SPM versus 26,121.8 mm^3^ for Analyze) and lower absolute values of maximum voxel intensity (3.7 ± 0.7 for SPM compared to 252.6 ± 2.8 for Analyze). These differences in cluster characteristics likely contributed to the discordance observed between the methods, as SPM tends to delineate more compact regions while Analyze identifies larger regions. Despite this, SPM demonstrated superior anatomical discrimination, particularly in cases of hippocampal sclerosis, further supporting its clinical applicability in patients with mesial temporal lobe epilepsy, where the epileptogenic zone occupies a smaller area.

Visual analyses of the images obtained from each method revealed that SISCOM performed using Analyze generates clusters with larger volumes and a higher number of clusters overall (an average of 14 clusters in Analyze versus 9 clusters in SPM). However, it was observed that these clusters often also include areas unrelated to the EZ, corresponding to regions of seizure propagation. This emphasizes the importance of establishing appropriate z-score thresholds, as discussed by De Coster et al. (22), who noted that lower thresholds increase the method’s sensitivity but may also include activation areas not directly related to the EZ.

There were some limitations in the present study. First of all, among the challenges of conducting a multicenter study is the difficulty in easily accessing some data. In particular, the injection time for patients from the Ribeirão Preto cohort was unavailable, limiting our ability to fully assess timing-related variability in SISCOM performance. As discussed by O’Brien et al., it is known that shorter intervals between the onset of the seizure and the administration of the radiotracer increase the accuracy in localizing the EZ. We found an unideal sensitivity in EZ localization with both tools, achieving 70%. This is slightly less than the literature which is around 92–93% in temporal lobe epilepsy (5, 13) and between 76–85% for refractory epilepsy in general (7, 17). This lower sensitivity may, in part, reflect longer delays between seizure onset and radiotracer injection in some cases. An additional limitation is the absence of EEG monitoring during interictal SPECT acquisitions, which may affect the ability to detect electrographic seizures that do not manifest clinically, especially in temporal lobe epilepsy. Furthermore, visual interpretation of the SPECT images was performed with access to clinical and EEG data. While this reflects standard clinical practice, it may have introduced bias in visual assessment.

Moreover, the small number of participants in the study limits definite conclusions. Only a few patients with postoperative Engel I-II outcomes from both institutions had the ictal and interictal SPECT available, resulting in a small number of participants in this study, consequently reducing the robustness of the statistical analyses. The numerical distribution from the statistical analysis indicates similar results for both methods, with comparable levels of agreement and disagreement with the reference standard. Consequently, the performance of the methods is similar; however, it is not statistically possible to determine which method is more accurate. Future prospective multicenter studies with standardized acquisition protocols, harmonized SISCOM analysis pipelines, and voxel-level comparison metrics are warranted to improve reproducibility, strengthen clinical reliability, and support broader adoption in surgical decision-making.

## 5 Conclusion

This study detected some technical differences between SISCOM using SPM and Analyze for the localization of the EZ in temporal lobe epilepsy. While Analyze presents larger and more numerous clusters, potentially increasing the possibility of encompassing the correct epileptogenic focus, it also includes seizure propagation regions and is lengthier. In contrast, SPM provides a refined anatomical delineation, particularly in cases with focal alterations such as hippocampal sclerosis, being faster and more feasible in clinical practice. However, despite these technical differences, both tools could localize EZ with similar sensitivity, suggesting that either can be effectively used to evaluate epilepsy. In this context, SISCOM-SPM offers the advantage of a semi-automated and examiner-independent workflow, reducing not only processing time but also improving reproducibility and usability in clinical settings. These qualities make it a particularly suitable option for pre-surgical evaluation in epilepsy centers, especially those seeking practical and accessible imaging solutions.

## Data Availability

The original contributions presented in the study are included in the article/supplementary material, further inquiries can be directed to the corresponding author.

## Footnotes

^1^Available from: http://www.fil.ion.ucl.ac.uk/spm/.
